# Cell-based technologies for Huntington’s disease

**DOI:** 10.1590/s1980-5764-2016dn1004006

**Published:** 2016

**Authors:** Mônica Santoro Haddad, Cristiane Valverde Wenceslau, Celine Pompeia, Irina Kerkis

**Affiliations:** 1 MD. Faculdade de Medicina da Universidade de São Paulo – Neurologia São Paulo, São Paulo, SP, Brazil.; 2 MD. Instituto Butantan - Genética, São Paulo, SP, Brazil.; 3 MD. Instituto Butantan - Genética, São Paulo, SP, Brazil.; 4 MD, PhD. Instituto Butantan - Genética, São Paulo, São Paulo, Brazil.

**Keywords:** Huntington's disease, stem cells, safety issues, cell therapy

## Abstract

Huntington's disease (HD) is a fatal genetic disorder, which causes the progressive breakdown of neurons in the human brain. HD deteriorates human physical and mental abilities over time and has no cure. Stem cell-based technologies are promising novel treatments, and in HD, they aim to replace lost neurons and/or to prevent neural cell death. Herein we discuss the use of human fetal tissue (hFT), neural stem cells (NSCs) of hFT origin or embryonic stem cells (ESCs) and induced pluripotent stem cells (IPSCs), in clinical and pre-clinical studies. The *in vivo* use of mesenchymal stem cells (MSCs), which are derived from non-neural tissues, will also be discussed. All these studies prove the potential of stem cells for transplantation therapy in HD, demonstrating cell grafting and the ability to differentiate into mature neurons, resulting in behavioral improvements. We claim that there are still many problems to overcome before these technologies become available for HD patient treatment, such as:

a) safety regarding the use of NSCs and pluripotent stem cells, which are potentially teratogenic;
b) safety regarding the transplantation procedure itself, which represents a risk and needs to be better studied; and finally
c) technical and ethical issues regarding cells of fetal and embryonic origin.

## Abstract (pt)

A doença de Huntington (DH) é uma desordem genética que provoca a destruição progressiva dos neurônios no cérebro humano. A DH deteriora progressivamente as habilidades físicas e mentais humanas, e é incurável. Tecnologias terapêuticas baseadas em células representam novas alternativas para diversas doenças neurodegenerativas, pois visam substituir neurônios e/ou prevenir a morte neuronal. Nesta revisão discutirmos o uso de tecido fetal humano, células tronco neurais (CTN) de origem fetal ou de células tronco embrionárias ou células tronco pluripotentes induzidas, em estudos pré-clínicos e clínicos. Além disso, o uso terapêutico de células derivadas de tecidos não-neurais, como células tronco mesenquimais, também será discutido. Todos estes estudos provam o potencial do transplante celular na DH, demonstrando a sua habilidade em enxertar no encéfalo e diferenciar em neurônios *in vivo*, resultando em melhorias comportamentais e motoras em modelos animais da DH. Nós também discutimos que há muitos problemas a serem resolvidos quanto à terapia celular na DH, tais como:

a) questões associadas à segurança do uso de CTNs, as quais são consideradas potencialmente teratogênicas;
b) segurança do procedimento de transplante intracerebral, que representa um risco ao paciente;
c) e, finalmente, questões técnicas e éticas associadas ao uso de células de origem fetal e embrionária.

## INTRODUCTION

American physician George Huntington first described the disease in 1872. Huntington's disease, which is also denominated Huntington's syndrome or Huntington's chorea, is a chronic, progressive and fatal neuropsychiatric disorder. HD is a hereditary autosomal dominant disease – 50% of children born from disease carriers inherit HD. This disease induces destruction of neurons in the subcortical parts of the brain hemispheres (mainly – striped body): mostly the caudate and lenticular nuclei. HD leads to the expansion of the anterior horns of the lateral ventricles - a sign used to identify HD during neuroimaging.^1,2^

Neuronal death in HD occurs as a result of abnormal synthesis of the Huntingtin protein (HTT), which is particularly prone to misfold and accumulates, leading to axonal and synaptic dysfunction. This accumulation of the misfolded mutant Huntingtin protein (mHTT) may affect intracellular transport of the protein or impair protein degradation by the proteasome, leading to autophagy.^3,4^ However, it is not clear how mHTT accumulates in the neuronal processes and induces an early pathological event in the brains of HD carriers.

There are two types of disease: adult-onset HD or juvenile HD, defined by the manifestations of the first signs of the disease. In adult-onset HD, the first symptoms usually appear after age 30, but possibly may start earlier.^3^ HD is characterized by a combination of motor and neuropsychiatric disorders that can occur simultaneously or in succession. Movement disorders include involuntary grimace, excessive gestures and others. Neuropsychiatric disorders, which usually appear after the motor disorders, may affect both cognitive and intellectual functions, as well as emotional status. Cognitive dysfunction is most often expressed in problems of visual-spatial orientation.^4,5^ Memory can also suffer, especially in the ability to retain the information necessary for workflow. Patients may not be able to plan and organize their activities. With time, symptom severity increases, reaching that of dementia. Emotional and behavioral disorders primarily present themselves by the emergence of unmotivated aggression and irritability (observed in more than half of patients).^6,4^ Approximately one third of patients suffer from depression. Remarkably, the cause of death among HD carriers is not the disease itself, but accompanying illnesses, such as pneumonia and heart failure; approximately 30% of patients die from suicide. Juvenile HD is a less common, early-onset form of HD that starts in childhood or adolescence. Juvenile HD evolves very rapidly and presents severe symptoms that quickly lead to disability.^7^

Currently, the diagnosis of HD is based on clinical (see above), genotypic and imaging findings. Since the discovery of the HD gene in 1993, a genetic test was developed that analyzes the HD mutation by counting the number of CAG repeats in the HD gene. This analysis enables direct confirmation of the diagnosis of HD in patients that exhibit HD-like symptoms. Using a blood sample, it is possible to demonstrate that an individual who does not have HD usually has 28 or fewer CAG repeats. HD carriers have 40 or more repeats, while a small proportion of individuals show a number of repeats that fall within a borderline region (Table 1).^8^

**Table 1:** Number of CAG repeats and HD outcomes that lead to HD development.

Number CAG repeats	Outcomes
≤28	Normal CAG number; individuals will not develop HD.
29-34	Risk for next generation, although individuals will not develop HD.
35-39	Risk for next generation. Some, but not all carriers will develop HD.
≥40	Individuals will develope HD.

Magnetic Resonance Imaging (MRI) is a neuroimaging modality that provides the greatest spatial and contrast resolution for assessing the type of lesion associated with HD and allows the most precise diagnosis. Magnetic resonance spectroscopy (MRS) can also be used to show impaired brain energy metabolism associated with, for example, increased regional brain lactate that plays an important role in the pathogenesis of HD. In addition, computerized tomography (CT) scan, which determines the damaged areas of the brain, is also useful in HD diagnosis.^9-14^

In conclusion, neuroimaging, particularly MRI, remains a keystone in diagnosing and assessing the severity of HD. Genetic testing is extremely important to confirm the diagnosis, especially if the family history is not forthcoming and, moreover, genetic counseling of HD patients may provide guidance on the implications of the disease.

No cure is currently available. All HD treatment focuses on symptom relief, thus achieving only temporary improvement of the patient's neurological status. Although such treatment improves patient quality of life, the progress of the disease is unchanged and the neuronal loss persists.

Neuronal cells and different types of stem cells are a promising raw material for the development of new therapeutic strategies in HD: they may prevent neuronal loss and consequently delay the disease progress.

## CELL-BASED THERAPIES

The main goal of cell-based technologies is to repair the mechanisms underlying disease initiation and progression, achieved by replacement of dead or defective cells and through the trophic effect, which some cell types may confer after their transplantation into the injured site.^15-17^ Different cell types can be utilized in these therapies, including fetal cells and tissues, progenitor cells or primary stem cells isolated from different tissues of an adult organism.^15,18-21^ This review covers the current state of cell-based therapies designed for HD. Some of these therapies are still at an initial stage of development and must be tested in animal models (preclinical studies) before they can be approved by regulatory agencies for use in clinical trials (involving humans). On the other hand, only a few studies have already reached clinical trials as will be discussed later.

## HUMAN FETAL TISSUE TRANSPLANTS IN HD PATIENTS

Human fetal tissue (hFT) has been used in basic research for decades.^18^ Clinical studies involving hFT aim to rebuild brain structures and neural circuitries by transplantation of hFT into the damaged central nervous system (CNS) in HD patients. The hFT is derived from elective surgical terminations of pregnancy in fetuses at between 6 and 12 weeks of gestation. In the case of the hFT used for clinical transplantation, the tissue consists of the whole ganglionic eminence, corresponding to the striatal primordium that ultimately develops into the caudate and putamen.^22-26^ HD patients may receive unilateral or bilateral hFT cell transplantation from several donated embryos (between five and nine).

Generally, the surgery and procedure of hFT transplantation into the brain of HD patients is considered to be safe.^22,26^ However, one study reported that three out of seven HD patients that underwent bilateral stereotactic transplantation developed subdural haemorrhages and two others required surgical drainage.^24^ No patients had adverse effects to the associated cyclosporin immunosuppression, nor did any patients exhibit deterioration following the procedure.^26^ The immunosuppressive protocol adopted in these studies efficiently prevents immune rejection of the graft. Histological evidence of immune rejection, including the appearance of microglia and macrophages in the hFT transplantation sites, has never been observed.^25,26^

All these studies show the ability of hFT cells to delay disease progression and provide stabilization or improvement in several neurological indices of cognitive functioning, although these changes were not uniform across HD patients.^22,24-26^

MRI analyses showed hFT graft survival and even growth without damage to the surrounding tissue.

Another key concern that needs to be considered is the safety of hFT transplantation regarding tumor formation.^27^ Although the majority of the studies do not show tumor formation after hFT transplantation,^26^ recently, one study emphasized the instability and risk of hFT grafts: the patient enrolled in a NIH-funded study did not show any behavioral improvement after hFT transplantation into the brain, yet presented a growth of tissue mass, termed "graft overgrowth".^28^ Such graft overgrowth may be explained by the presence of immature neuroepithelial cells actively dividing cells that express NSC markers such as Sox2, which are occasionally preserved in the hFT-derived cell suspension used for transplantation.^28^ Both these studies^26,28^ compromised the safety of hFT transplantation and indicate the need for further in-depth studies using chemical and transgenic preclinical models and a large number of animals. Additionally, it has been shown that not only hFT transplantation, but also fetal stem cell transplantation, may sporadically lead to brain tumor formation at the site of transplantation.^29^ Although these cells are subject to restrictions regarding safety, they are able to recover medium spiny neurons (MSN).

A postmortem analysis of a brain carried out six months after a patient underwent transplantation demonstrated that hFT-derived cells were able to differentiate into MSNs which expressed dopamine-receptor related phosphoprotein (32 kDa) (DARPP-32), the neuronal nuclear antigen (NeuN), calretinin that can protect the MSNs against neurodegeneration in HD, and somatostatin, which is increased in the basal ganglia in HD.^20,28^ These results are important given that HD is considered a disease of the striatum, characterized by vulnerability to degeneration and death of MSNs.^1,2^ Also, hFT graft-derived astrocytes have also been observed after transplantation.^20^ Astrocytes are a sub-type of glial cells in the CNS that are protective neurons against excitotoxicity by removing excessive glutamate from the extracellular space. Astrocyte recovery after hFT transplantation is a highly beneficial effect of the procedure, since in the HD brain, where mHTT accumulates in glial nuclei, there is a decrease in the expression of glutamate transporters in neurons and astroglial cells.^30^

Misfolded mHTT protein aggregates, which accumulate as cytoplasmic aggregates and nuclear inclusions, are a hallmark feature of HD.^3,4^ As we mentioned above, mHTT aggregates clump together and damage neurons. Recent research suggests that these mHTT proteins may also be transmitted from neuron to neuron. Interestingly, this hypothesis of pathogenic protein spread during neurodegeneration has been critically evaluated recently by Walsh,^31^ who suggests experimental approaches to rigorously test its fundaments. However, the formation of mHTT aggregates does not seem to be a prerequisite for HD, as shown by a clinical study carried out with transplanted hFT in HD patients, whereby a postmortem histological analysis performed 18 months after the treatment did not show the presence of neuronal protein aggregates of mHTT within the hFT graft.^32^ In this context, all experimental data that support or detract from this hypothesis are of great importance.

## ANIMAL MODELS OF HD

To date, of the possible transplant options that can benefit HD patients, only hFT has been used in clinical HD studies. Thus, for the purposes of the present review, we have provided a short explanation about animal HD models, which are most commonly used in preclinical studies for other treatment approaches. Preferably, the HD animal model should provide similar genotypes and phenotypes as human HD.^15,33^ Chemical HD animal models are commonly induced by treatment with quinolinic acid (QA)^19,34,35^ or 3 nitropropionic acid (3-NP)^36-39^ Both of these chemical agents induce rapid development of progressive HD, early disease onset, abnormal behavior, and neuropathological features. These models are useful for rapid evaluation of the therapeutic hypothesis (acute models).^15^ In contrast, transgenic models are providing more precise systems for disease replication that allow evaluation of the therapy of interest in slow progressive degeneration.^40^

**Figure 1:**
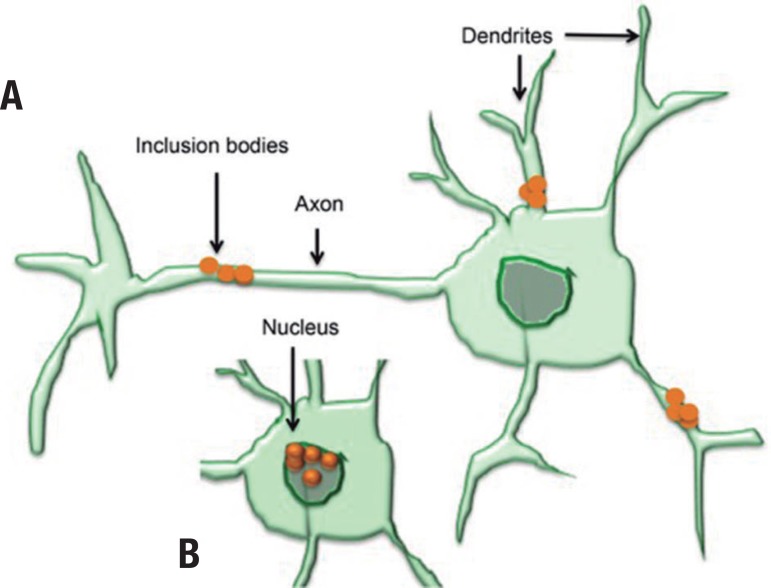
Aggregate formation by HTT mutation in neuron cell derives inclusion bodies: [A] Inclusion body accumulation at axons and dendrites (cytoplasm); and [B] nucleus. Black arrows indicate inclusion bodies.

Transgenic models result from the random insertion of a portion of the human *HTT* gene containing the coding region for the polyglutamine repeat into the mouse genome, under control of several promoters. The most commonly used mouse HD models are R6/1, R6/2 and N171-Q82, which express truncated N-terminal fragments.^41^ Moreover, in contrast to the chemical models, the transgenic HD models are powerful research tools because they recapitulate specific HD features such as the accumulation of intracellular aggregates of mHTT in the cytoplasm or nucleus of neurons, known as inclusion bodies (IBs) (Figure 1).

Rodent HD models such as rats and mice are widely used to investigate cell-based therapy technologies.^40^ More recently, a transgenic nonhuman primate model in rhesus monkeys (*Macaca mulatta*) of HD was developed.^42^ Also, transgenic HD models were established in large farm animals such as sheep (*Ovis aries*)^43^ and Tibetan miniature pigs.^44^ However, transgenic HD models in large animals are high cost and technically difficult to use in routine cell-based therapy testing.

## NEURAL STEM CELL TRANSPLANTATION IN HD ANIMAL MODELS

**Figure 2:**
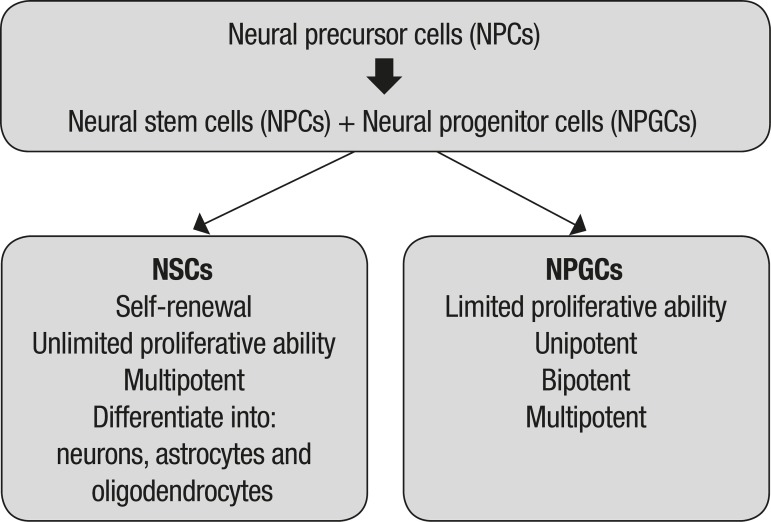
Hierarchical relationship between types of brain-derived stem cells.

It was long thought that neural cells could not undergo regeneration in injured or diseased human brain. However, advanced studies with NSCs in injured animal models have shown the regenerative potential of these cells.^15,45,46^ NSCs are self-renewing cells able to generate neurons, glial cells and astrocytes. NSCs can be derived from embryonic, fetal or adult tissues.^47,48^ Embryonic-fetal neural precursor cells (NPCs) are composed of a mixed population of multipotent NSCs and transit amplifying/intermediate progenitor cells (NPGCs) (Figure 2).^49^

Discrete populations of NSCs can be found in specific areas of the postnatal human brain and seem to play an important role in postnatal growth as well as in recovery of neural tissue from injury, anoxia, or disease.^50^ NSCs can be isolated at different stages of development and cultivated *in vitro* under appropriate culture conditions as neurospheres, which are free-floating clusters of round multicellular spheroids, or, alternatively, as adherent NSCs (Figure 2). Neurospheres, when allowed to adhere to the substrate, start to produce neural rosettes (Figure 2), which are long-term self-renewing neuroblast-like cells. Murine or human NSCs have been used in preclinical studies to show the potential therapeutic effect of NSC-based technologies on neurogenesis in HD animal models.^34,51,52^ These studies showed that transplantation of NSCs improves motor function, extends life span and even lessens mHTT intracellular aggregate formation. In these studies, the cells were injected mainly by the intrastriatal route due to the inability of NSCs to pass though the blood brain barrier (BBB). NSCs exhibit robust engraftment at the site of injury. However, the differentiation of these cells into neural cells is still controversial.^34,37,38,51-53^

The evaluation of teratogenicity of embryo-fetal derived NSCs is still lacking, although it is essential for the further translation of these studies into clinical trials. Due to the commitment of NSCs to neural fate, they may be considered an ideal cell type for the treatment of neurological diseases. However, NSCs are difficult to obtain in therapeutically significant quantities and pose serious ethical and religious limitations given that they are obtained from aborted fetuses.^15^

## HFT AND NSC FROM HFT TRANSPLANTATION AND ETHICAL CONCERNS

There is no doubt that hFT and NSCs are important in many research areas, especially in studies of human neurodegenerative diseases. HFT and NSCs help scientists investigate many aspects of basic science that cannot be studied in any other way. However, isolation of hFT and NSCs from hFT raises major ethical, political and religious controversies.^21,54^ We believe that these ethical issues, associated with the limited number of experiments carried out so far, only minor success in these experimental therapies, as well as the potential teratogenicity of fetal and embryonic cells, are reasons why the use of ethically-accepted alternatives is starting to dominate the field, especially in Brazil.

## PLURIPOTENT STEM CELL TRANSPLANTATION IN HD ANIMAL MODELS

**Figure 3:**
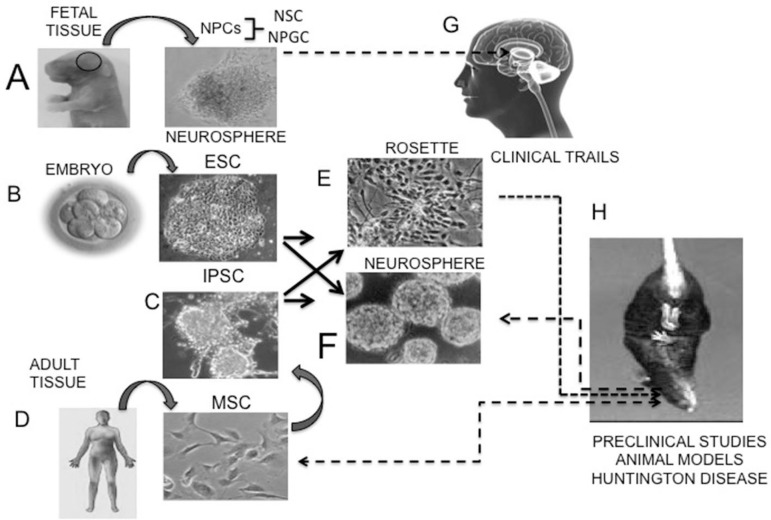
Stem cell types used so far in clinical and preclinical studies of HD. [A] hFT is isolated from fetuses at between 6 and 12 weeks of gestation and composed of NPCs (NSC+NPGC) and many other fetal cell types that are used in clinical studies (G). Similarly, hFT, after further purification, can be used as a source of NSCs, which, in turn, are also used in clinical studies (G). [B] ESCs can be isolated from early human embryos (B), and form rosette colonies (E) and neurospheres (F). Both rosette- or neurosphere-forming NSCs (A, B) have been used in preclinical studies (H). [C] MSCs are isolated from bone marrow, adipose tissue or umbilical cord and have a different morphology *in vitro* than that of NPCs or ESCs, being fibroblast-like (C). [D] iPSCs can be obtained from many adult tissues (C) via reprogramming; these cells are morphologically similar to ESCs (B). However, unlike ESCs, iPSCs are obtained from adult cells and therefore are not subject to the same major ethical considerations as those for ESC isolation, which require human embryos; iPSCs also produce NSCs rosette-forming colonies (E) or neurospheres (F), which have been used in preclinical studies (H).

Embryonic stem cells (ESCs) are pluripotent cells isolated from the inner cell mass of early embryos. These cells can generate a whole organism when re- introduced back into the embryonic environment (e.g. blastocysts) (Figure 3). ESCs are also able to form gametes – reproductive cells. Additionally, ESCs form noncancerous tumors called teratomas, which is one of their fundamental traits.^55,56^

As mentioned before, tumor formation is a major safety concern for those who hope to use the cells therapeutically.^57^ Due to this feature of ESCs, they cannot be used unspecialized in preclinical and clinical studies. To analyze the therapeutic potential of human ESCs in HD animal models, they have been reversed to NPCs that have been transplanted directly into the striatum of the animal models. Different types of NPCs may be obtained, which depends on the protocol of human ESC differentiation.^58-60^ Rosette-forming ESC–derived NPCs (Figure 3) are unable to differentiate *in vivo* into medial spiny neurons (MSN), which is a principal target of HD therapies.^59^

However, they have the capacity to differentiate into other neuron types and into astrocytes. Preclinical short-term studies show effective recovery of motor deficit after hESC-derived NSC transplantation in the QA rat model.^58-60^ The main problem in using ESC-derived NPCs in the clinic is the need to control their proliferation in order to avoid neural cell graft overgrowth.^59,60^

Laboratory-grown induced pluripotent stem cells (iPSCs) are a type of pluripotent cell that can be generated directly from adult cells (Figure 3). Currently, practically any type of somatic adult cell can be reprogrammed back into an embryonic-like pluripotent state. The iPSCs share all principal characteristics of ESCs, having the ability of long-term self-renewal, maintaining an unspecialized state; and giving rise to (under specific treatments) specialized cell types and teratomas when injected *in vivo.*^61,62^ However, the pluripotential ability of the iPSCs may be influenced by the adult donor cell source.^63,64^ The therapeutic potential of hiPSCs, as well as that of hiPSC-derived NPCs, has also been investigated using HD animal models.^65-67^ These cells have been transplanted into both chemical^65^ and transgenic HD rodent models via the ipsilateral ventricular route.^67^ These cells graft into recipient brains and differentiate into GABAergic MSNs^65,67,68^ and astrocytes.^68^ After transplantation, a modest reduction in striatal neuronal atrophy, a hallmark of HD disease that appears before the onset of motor symptoms, can be observed.^68,69^

After transplantation of NSCs derived from iPSCs, short- and medium-term functional motor improvements have been documented in comparison with sham group animals.^65,67,68^ However, long-term motor functional recovery from HD followingESC- and iPSC-derived NSC transplantation still needs to be further evaluated.

## MESENCHYMAL STEM CELLS IN HD ANIMAL MODELS

MSCs can be found in virtually all postnatal tissues, including the embryonic annexes (umbilical cord and placenta), bone marrow and adipose tissue. Isolation of these cells is associated with fewer ethical concerns when compared with ESCs. MSCs have a fibroblast-like morphology (Figure 3), express a set of specific markers and adhere to plastic. They are of exceptional interest because they are unspecialized cells capable of self-renewal and differentiation into specific cell types, especially into mesoderm derivatives.^70-73^ However, a growing number of studies has demonstrated their capacity to also differentiate into neural cells.^74^ Furthermore, the number of clinical trials using MSCs is rising every year and, at least, the clinical safety of these cells has been confirmed.^75^

The biological function of MSCs is to replace tissues in response to normal cellular turnover or trauma.^76^ It has been also suggested that these cells can be recruited to nascent microvascular walls during development and postnatal growth.^77^ Accordingly, MSCs in their anatomical sites – stem cell niches – have their growth arrested until they are triggered to restart proliferation and even differentiation in response to physiological cues, such as tissue turnover or repair, or experimentally, when isolated and cultivated *in vitro*. In bone marrow, for example, subendothelial osteoprogenitors become stem cells acting as pericytes in different postnatal tissues. Therefore, as an alternative to differentiation, tissue-specific MSCs may function to support the regeneration of other local cell types. Such support is carried out by MSCs after transplantation into the unhealthy organism via the secretion of a variety of bioactive molecules such as cytokines, which have 'trophic activities' that can promote a regenerative microenvironment.^16,17^ MSCs also benefit tissue reconstruction by stimulating angiogenesis, the production of immunomodulatory mediators and even by delivering other molecules to the injured site. MSCs also act by reducing chronic inflammation, inhibiting apoptosis and decreasing scar formation. They are able to stimulate mitosis of tissue-intrinsic stem cells and reduce the destructive effects of oxidative stress.^16,17,78^ MSCs express very low levels of MHC class I proteins and lack MHC class II proteins, and can therefore be transplanted into other organisms of the same or different species without rejection.^73,79^

MSCs isolated from bone, adipose tissue and umbilical cord have been used in different chemical (QA and 3-NP) and genetic animal models (R6/2-J2, N171-82Q, R6/2) of HD, which were transplanted using the intracerebral route (directly into the striatum). These studies demonstrate that MSC transplantation leads to behavioral and memory improvements, reduced brain damage, improvement of striatal degeneration, and enhanced expression of several striatal growth factors, which are attributed to the neuroprotective effect of MSCs. MSC transplantation shows robust cell engraftment at the site of lesion, as well as the ability of these cells to migrate to adjacent areas.^80-83^ Although the actual differentiation of MSCs into neurons is controversial, it is not required to justify the beneficial activity of these cells in HD. Furthermore, MSC transplantation has never been shown to lead to teratoma or graft overgrowth formation, indicating the safety of these cells, at least in animal models.

## NEUROTROPHIC FACTORS

Neurotrophic factors, such as brain-derived neurotrophic factor (BDNF), are essential contributors of CNS neuron function. Studies demonstrate their reduced availability in diseased brains, thus suggesting that they play an important role in neurological disorders and, in particular, in HD.^84,85^ Under non-pathologic conditions, BDNF is synthesized in the cortex, the substantia nigra pars compacta, the amygdala, and in the thalamus. All these regions supply the striatum with BDNF.^86,87^ In HD, the deficit of BDNF in the striatum may be due to reduced BDNF gene transcription in the cerebral cortex or reduced BDNF vesicle transport (or both).^88,89^ The decrease in BDNF expression observed in HD impairs dopaminergic neuronal function, which may be associated with HD motor disturbances. As a result, many studies have been carried out to examine whether increasing BDNF levels may help treat HD.^37,83,88^

NPCs and MSCs, besides their differentiation ability to produce neurons, have been extensively investigated with respect to neurotrophic factor secretion.^37,90^ Studies have shown that MSCs derived from adipose tissue and bone marrow are able to secrete BDNF *in vitro.*^83,91-93^ However, MSCs secrete BDNF at low levels, which are dependent on tissue source used for MSC isolation and on donor characteristics.^92,94,95^

Therefore, recent studies have generated NSCs derived from ESCs that overexpress BDNF in order to evaluate whether they have enhanced therapeutic abilities in HD. Neurons derived from BDNF-GFP-expressing ESCs harbor a more complex dendritic morphology and differentiate into the GABAergic lineage.^96^

## CONCLUSION

Great advances have been made in cell-based therapies over recent decades and it is expected that, in the future, such therapies will be provided alone or in combination with traditional medication, new small molecule drugs and biological drugs to patients who suffer from neurological conditions such as HD. Figure 2 summarizes the major advances that have been made in cell-based technologies and their use for the treatment of HD in clinical and preclinical studies.

Notably, all these cell-based technologies may be roughly divided into two: NSC- and MSC-based, since none of the pluripotent cells (such as ESCs or IPSCs) can be used intact/unspecialized due to the risk of teratogenicity after transplantation into the lesion site. NSCs derived from iPSCs look set to substitute hFT, fetal- as well as ESC-derived NSCs, thus avoiding ethical and religious considerations.

Another important conclusion is that these different cell types produce very similar clinical outcomes and therefore can be used interchangeably. The major advantage of MSCs over NSC derived from different cell types is that they do not produce teratomas or graft overgrowth. Additionally, they express neurotrophic factors, albeit at low levels, which are apparently important for HD improvement.

The major problem of cell-based technologies in HD concerns the route of administration, which is usually intracerebral. This will be a great obstacle for the use of cell-based technologies in HD patients, which is a genetic disease and may require more than one cell transplantation. None of the clinical and preclinical HD studies have yet used the systemic intravenous route,^21,24,26,27^ probably due to the fear that these cells will not penetrate the BBR in significant numbers. However, we believe that MSCs can be systemically administrated and have tropism for sites of injury, even the brain, as in the case of HD. However, further studies are necessary to better understand this administration route and prove its efficacy and safety in HD.
